# Accentuation and Attention: From Perceptual Organization to Consciousness

**DOI:** 10.3390/brainsci15030243

**Published:** 2025-02-25

**Authors:** Baingio Pinna, Daniele Porcheddu, Jurģis Šķilters

**Affiliations:** 1 Department of Biomedical Science, University of Sassari, 07100 Sassari, Italy; 2 Department of Economics and Business, University of Sassari, 07100 Sassari, Italy; daniele@uniss.it; 3 Laboratory for Perceptual and Cognitive Systems, Faculty of Science and Technology, University of Latvia, LV-1586 Riga, Latvia; jurgis.skilters@lu.lv

**Keywords:** visual attention, visual illusions, perceptual organization, dissimilarity, accentuation principle

## Abstract

**Background**: This study investigates the complex relationship between accentuation and attention in visual perception, extending classical Gestalt principles by introducing dissimilarity as a complementary mechanism to similarity in perceptual organization. **Objectives and Methods**: Through a series of phenomenological experiments, we demonstrate how accentuation, driven by dissimilarity, plays a crucial role in shaping visual experience and guiding attention. **Results**: Our findings reveal that accentuation serves as a pre-attentive mechanism for highlighting salient features, influencing initial perceptual organization, and modulating the apparent shape and orientation of visual elements. We show that while accentuation operates rapidly and automatically, attention acts as a flexible, selective mechanism that can either reinforce or override accentuation-based percepts. This interplay suggests a two-stage process of visual perception, with implications for theories of consciousness and information processing in biological systems. This study also explores the evolutionary significance of accentuation in camouflage and sexual selection, providing insights into how perceptual mechanisms may have evolved to enhance adaptive fitness. **Conclusions**: Our results have broad implications for understanding visual cognition, design, and clinical applications related to attentional disorders.

## 1. Introduction

The interplay between camouflage and accentuation in biological systems offers profound insights into the evolution of visual perception and attentional mechanisms. While the camouflaging function of leopard spots is well documented [1], other organisms utilize similar patterns to accentuate specific body parts while concealing others. This phenomenon is particularly evident in Lepidoptera and various aquatic species, where ocelli (eyespots) serve to highlight non-vulnerable anatomical regions [2].

Ocelli, although not functional eyes, have evolved to fulfill multiple adaptive purposes, significantly enhancing the survival prospects of their bearers in natural habitats. The primary function of these structures is predator deterrence [3]. When a potential predator approaches, the sudden display of eyespots may simulate the presence of a larger organism, inducing a startle response in the predator and providing the prey with a critical temporal window for escape (on the neural basis of such rapid decisions see [4,5,6]). Avian predators, in particular, demonstrate susceptibility to this deceptive mechanism [7].

The strategic positioning of eyespots on less vital anatomical regions, such as wing tips, represents an evolutionary strategy to redirect predatory attacks away from critical areas like the head or thorax [8]. This adaptive placement allows for potential survival even in the event of partial wing damage. Moreover, ocelli contribute to Batesian mimicry, a phenomenon where harmless species evolve to mimic the aposematic signals of more dangerous organisms [9,10].

While less prevalent, there is empirical evidence suggesting that ocelli may play a role in sexual selection in certain species. These markings potentially serve as indicators of genetic quality, with larger or more symmetrical eyespots potentially signaling superior fitness to potential mates [11].

Paradoxically, despite their conspicuous nature, eyespots can, in specific environmental contexts, contribute to camouflage. When organisms with ocelli rest in naturally spotted environments, such as foliage with water droplets or in dappled light conditions, the eyespots can disrupt the organism’s outline, reducing its detectability to predators [9,12,13].

The evolution of ocelli exemplifies the capacity of natural selection to exploit diverse ecological interactions. This phenomenon involves the accentuation of one anatomical region to obscure another, simultaneously attracting and diverting predator or prey attention. Such mechanisms hyperstimulate the inherent tendency of attention to be drawn toward dissimilar, highlighted, or accentuated elements [9,12]. The salience of the accent correlates positively with the intensity of attention directed towards it and inversely with the ability to voluntarily divert attention from the attracting stimulus (see also [14]).

These evolutionary processes and resultant phenotypic patterns, aimed at enhancing adaptive fitness, culminate in a sophisticated interplay of deception, illusion, accentuation, and camouflage that modulates attention. The fundamental biological imperative of detecting salient, survival-critical information, even when obscured, masked by noise, or camouflaged, aligns closely with the core function of attention [15]. In this context, relevant information for an organism encompasses the set of meaningful signals necessary for its biological functions.

The complex dynamics of predator–prey interactions hinge on the strategic deployment of camouflage and accentuation, leveraging innate tendencies and laws of perceptual organization and attention. This intricate interplay of deception and illusion likely constitutes a principal mechanism driving natural selection and environmental adaptation [16].

This paradigm implies that organisms evolve strategies to increase uncertainty and minimize or eliminate information that could compromise their survival. Concurrently, they enhance or emphasize irrelevant information, generating false signals and diverting the attention of potential threats. In reproductive contexts, such as floral displays or mating behaviors, the objective shifts to amplifying relevant information to compete more effectively with conspecifics and achieve reproductive success [17]. In essence, all living organisms can be conceptualized as entities that generate, detect, and manipulate information, leading to the complex interactions fundamental to their survival. This perspective aligns with the broader framework of information theory in biological systems [18].

This analysis gives rise to several key concepts central to this work: the perceptual notions of accentuation and attention. These concepts prompt critical questions: What is the nature of accentuation? How does attention influence perceptual organization in the context of accentuation and camouflage? What is the relationship between accentuation and information? To what extent are perceptual organization and attentional mechanisms related? (For a prior overview of the relevant research, see, e.g., [19,20,21]).

Furthermore, the concept of illusion and illusoriness associated with accentuation and camouflage warrants consideration, as it influences the perception of ambivalence, uncertainty, awareness, perceived danger, and objective reality. This, in turn, affects the ability to discern the veracity of perceptions and the perceptual process itself. These considerations lead to further inquiries: How does species evolution manipulate the principles of perceptual organization and attentional biases to deceive other organisms while simultaneously evolving mechanisms to avoid deception? Addressing these questions necessitates a comprehensive investigation into the concepts of accentuation, camouflage, information processing, perceptual organization, attention, epistemological truth, and ultimately, the nature of consciousness and unconsciousness.

This work aims to address these questions through a detailed phenomenological study of extreme conditions in perceptual organization, facilitating reflection on the relationships between the key concepts of accentuation and attention. Accentuation will be explored in several potential meanings and phenomenological applications, providing a foundation for understanding its role in visual perception and cognition.

## 2. Materials and Methods

### 2.1. Participants

Unless otherwise specified, each experiment outlined in the following sections involved distinct groups of 20 undergraduate students from diverse disciplines, such as linguistics, literature, human sciences, architecture, and design of the University of Sassari, Italy. The participants had limited knowledge of Gestalt psychology and were unaware of the specific phenomena being investigated or the experimental objectives. The groups contained both male and female students (50%), all with normal or corrected-to-normal vision.

### 2.2. Stimuli

The stimuli were presented on a 21″ color CRT monitor (Sony GDM-F520, 1600 × 1200 pixels, 100 Hz refresh rate) (Sony Corporation, Tokyo, Japan) controlled by a MacBook computer. The testing environment was illuminated with an Osram Daylight fluorescent light (250 lx, 5600 K) (OSRAM Licht AG, Munich, Germany). The stimuli were generated using a robust yet straightforward graphic design tool like Adobe Illustrator (v. 28.4.1), making them easily replicable. All stimuli were displayed on a frontoparallel plane positioned 50 cm from the observer. Participants’ head positions were stabilized using a chin rest, and stimuli were viewed binocularly.

### 2.3. Procedures

The experiments primarily employed a phenomenological free-report method, where participants were asked “What do you see?” Each group of 20 observers described a single stimulus from the stimuli sets presented in the following sections, ensuring no cross-stimulus interaction or contamination. The descriptions included in the study reflect spontaneous reports from at least 16 out of 20 participants in each group, with edits made for brevity and clarity. To maintain objectivity and prevent interpretive bias, three independent linguistics graduate students, who were unaware of the study’s hypotheses, reviewed the descriptions [22,23,24,25,26,27]. These descriptions were then incorporated into the analysis to support the arguments being made. The reports were spontaneous, and the experiment concluded once participants had completed their descriptions. Observation time was not limited, and participants could view the stimuli throughout their reporting (see also [28,29,30,31,32,33,34]).

For simplicity, and to avoid weighing down the flow of reading and comprehension, we report below the data that are representative on average of all the phenomena described in the subsequent sections.

A total of 16 out of 20 participants (80%) reported experiencing the described illusory effect. The estimated proportion was therefore *p =* 0.80. The corresponding standard error was approximately 0.089. A 95% confidence interval for the proportion, based on the normal approximation ranged from approximately 0.63 to 0.97.

To assess whether this proportion significantly differs from chance level (e.g., 50%), we conducted an exact binomial test. The test indicated a statistically significant deviation from 0.50 (*p* < 0.01). Thus, the observed proportion of participants reporting the illusory effect was significantly higher than the hypothesized baseline.

All participants (N = 20) provided an illusion intensity rating on a 0–100 scale. Ratings were tightly clustered at the high end, with a mean of 93.65 (SD = 3.57), a median of 93.5, and a range of 88 to 100. A one-sample t-test against 50 indicated that ratings were significantly higher than chance-level assumptions, t(19) = 54.56, *p* < 0.0001, 95% CI [91.98, 95.32]. The effect size was exceptionally large (Cohen’s d = 12.23), confirming that participants perceived a robust illusory effect.

During the experiments, participants were encouraged to reflect on what they were observing and to explore the stimuli from multiple perspectives. The experimenter asked additional questions to prompt deeper and more detailed observations. Any variations that arose during this exploration were documented by the experimenter and are recorded in the subsequent sections.

## 3. From Perceptual Grouping to Accentuation

Gestalt psychologists ([31,35,36]; for an overview, see also [37]) discovered the fundamental principles of perceptual organization, which have become cornerstone concepts in visual perception research. These principles include proximity, similarity, continuity, closure, symmetry, convexity, Prägnanz (simplicity), past experience, common fate, and parallelism. The proximity principle, in particular, posits that all else being equal, spatially contiguous elements are perceptually integrated into a unified group.

**Figure 1:**
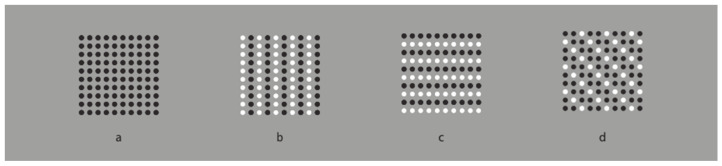
Proximity and similarity principles: all else being equal, the closest and most similar elements are grouped together creating columns (**a**,**b**), rows (**a**,**c**), and oblique arrangements (**d**).

Figure 1a exemplifies this principle, depicting a matrix of dots arranged along orthogonal axes. The equidistant spacing facilitates bistable percepts oscillating between row and column organizations, engendering a stable perception of uniformity. The high degree of homogeneity elicits the perception of a continuous dotted surface, despite the discrete nature of the individual elements. At a primary phenomenological level, the dots are perceived as constituents of a matrix, while at a higher-order level, they coalesce into a dotted square surface. Alternative organizational structures, such as zigzag patterns or diagonal groupings are perceptually and cognitively demanding, even with directed attention, as proximal elements exert a stronger influence on visual organization [38].

To perturb the stability of the percept while preserving the proximity principle, dissimilarity can be introduced between rows and columns. Figure 1b,c demonstrate differential grouping achieved through contrast polarity reversal [39,40]. Under these conditions, observers report perceiving alternating columns or rows of achromatic dots. In Figure 1d, diagonal grouping is accentuated through strategic manipulation of contrast polarity. This novel grouping mechanism operates independently of the proximity principle, as the inter-element distances along the diagonal exceed those along the cardinal axes. Wertheimer [35,36] attributed these groupings to the similarity principle, which states that all else being equal, perceptually similar elements tend to be integrated into a cohesive group.

The homogeneity observed in Figure 1a is in this way disrupted in subsequent iterations, allowing for the emergence of novel information along the induced groupings. Attentional modulation is facilitated by the ability to shift focus between columns or rows. Compared with Figure 1a, it becomes increasingly challenging to attend to groupings that contradict the similarity-based organization, such as diagonal arrangements, or to perceive rows where columns are dominant, and vice versa. Notably, even in Figure 1a, where proximity-based grouping primarily yields rows and columns, the introduction of dissimilarity significantly enhances the salience of these linear structures. Consequently, while in Figure 1a these structures may not be fully accessible to consciousness, in the subsequent figures, they become the primary perceptual focus, relegating the homogeneous global form to the background, resulting in its fragmentation.

This phenomenon is analogous to certain forms of biological camouflage, such as the disruptive coloration observed in zebras [41,42]. The alternating black and white stripes serve to fragment the zebra’s outline, impeding predator recognition of individual targets, particularly in group contexts or in environments characterized by high visual complexity, such as tall grasslands or during locomotion [43].

At first glance, Wertheimer’s explanation for how humans describe perceptual experiences appears simple, highly convincing, and well supported by empirical evidence [44,45,46,47,48,49,50].

Expanding upon prior phenomenological observations, a critical insight emerges: the introduction of dissimilarities through contrasting elements disrupts the homogeneity of proximities among rows and columns, thereby inducing dominance of one configurational percept over another. Concurrently, similarity in contrast polarity between dots facilitates their integration into coherent perceptual units. This interplay between dissimilarities and similarities serves dual functions: dissimilarities aid in segmenting and differentiating adjacent groups, while similarities enhance intragroup cohesion [51,52,53]. Dissimilarities start at a different level or perceptual organization that dominates the other ones.

In this context, dissimilarities and similarities operate synergistically, augmenting perceptual grouping by accentuating intergroup differences and reinforcing intragroup homogeneity. This process can be conceptualized as two complementary dynamics: dissimilarities functioning as demarcation lines that emphasize discontinuities within the visual field, and similarities reinforcing internal consistency, thereby enhancing the salience of segregated objects [52]. Fundamentally, dissimilarities delineate and isolate the boundaries of emergent forms, while similarities consolidate the perceived uniformity of their internal structures [12,54,55].

This segmentation process, primarily driven by dissimilarity, introduces discontinuities in the visual field, generating novel information by reducing uncertainty. Consequently, attention is naturally drawn to this emergent information, rendering alternative groupings that deviate from the established perceptual organization highly improbable. In essence, dissimilarities function as potent attractors for bottom-up attention, serving as salient features that capture and direct focus.

The interaction between similarity and dissimilarity in Figure 1, as anticipated by Wertheimer, results in the perception of rows, columns, or diagonals. However, the phenomena extend beyond these primary percepts. Figure 1b,c encompass additional perceptual effects that are not immediately apparent and can only be discerned through focused attention. This process involves bringing into conscious awareness elements that are initially obscured within the array of points, particularly the differentiation of columns and rows as distinct perceptual entities.

**Figure 2:**
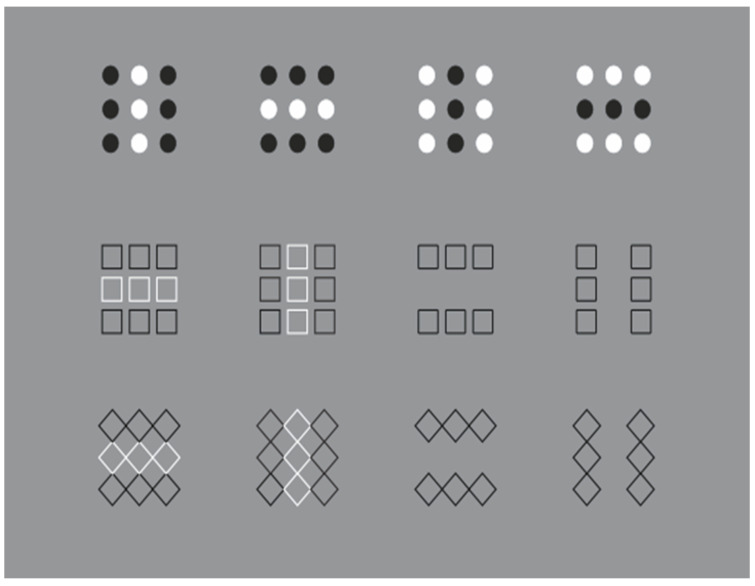
Despite the shape of each pattern having a geometrical square shape, it appears as a vertical or horizontal rectangle following the directions of the grouping.

Upon re-examination of Figure 1b,c, focusing on 3 × 3 sub-matrices within each global matrix and engaging in a non-immediate perceptual process, a phenomenon of apparent elongation and widening becomes evident, aligning with the direction of grouping, as reported by our participants. These observations suggest that perceptual organization extends beyond the simple grouping principles proposed by Wertheimer, giving rise to additional perceptual phenomena [56]. To better visualize these illusory effects, one can examine the geometric square patterns in Figure 2, which exhibit more pronounced elongation or widening depending on the grouping direction (cf. [12,23,24,54,55]).

**Figure 3:**
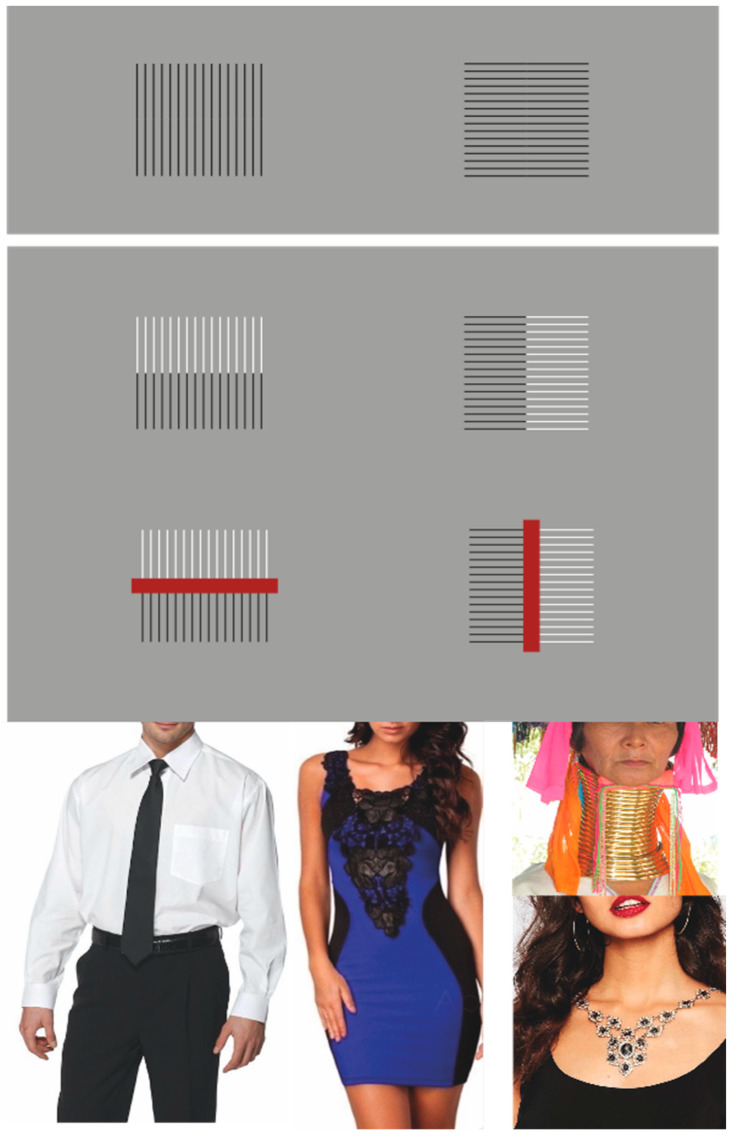
Helmholtz square illusion. More pronounced effect of elongation or widening than those observed in both Figure 2 and the Helmholtz square illusion. The tie effect (human pictures are sourced from publicly available sources).

This outcome appears to contrast with the well-known Helmholtz square illusion (Figure 3—first row), where a square composed of horizontal segments is perceived as taller and slimmer, while the same square made up of vertical segments appears wider, resembling a horizontal rectangle [57]. In essence, a square with horizontal segmentation is perceived as a vertical rectangle, while one with vertical segmentation appears as a horizontal rectangle.

However, upon re-examining the conditions shown in Figure 2, we observe an apparently opposite effect: vertical groupings create a vertical rectangle, while horizontal groupings produce an illusory horizontal rectangle. At first glance, this seems to present an irreconcilable contradiction between the two cases. Nevertheless, this contradiction is only superficial. The perceived shape is not solely determined by the orientation of individual segments in the Helmholtz square but rather by the overall arrangement of elements that holistically determines the form. This principle holds true regardless of whether the elements are segments, dots, squares, or diamonds. Thus, because the horizontal segments in the Helmholtz square are arranged vertically, the result is a perception of a vertical rectangle, while the opposite occurs with vertical segments arranged horizontally. In short, it is the arrangement of the elements, whether stacked or juxtaposed horizontally, that determines the overall shape of the elements. This accentuation in the element organization parallels the conditions illustrated in Figure 2, solving the contradiction.

There is a common misconception that shirts with horizontal stripes make the body appear wider and heavier, while vertical lines have a slimming effect. In fact, the opposite is true, as demonstrated by the Helmholtz square illusion [58]. However, this belief is partially validated when considering the groupings of elements like those illustrated in Figure 2. It is plausible that the horizontal stripes present on the rear (hindquarters) of prey species such as zebras and okapis serve to create an illusion of increased size in that area, which is typically the initial target for predators [42].

The fact that there is a common misconception about the stripes on shirts does not mean that we do not perceive them in accordance with the described illusions. In general, we can assert that the perceptual and cognitive levels may, but do not necessarily, act in concert. They can also contradict each other and proceed in opposing directions, creating paradoxes (cf. [12]). In this sense, studying accentuation becomes a useful tool for understanding the potential discrepancy between these two levels of processing, and, consequently, it allows us to likely derive different principles.

If this principle holds true, we can hypothesize the production of an even more pronounced effect of elongation or widening than those observed in both Figure 2 and the Helmholtz square illusion. This enhancement can be achieved departure from squareness, as illustrated in Figure 3—second row. This figure presents two conditions where the horizontal and vertical orientations and arrangements have been duplicated. In the left condition, two horizontal directions are accentuated: one due to the arrangement of individual vertical segments, and another due to the two horizontal bands formed by black and white vertical segments. Conversely, in the right condition, vertical directions are emphasized. This configuration produces a significantly more intense phenomenon of illusory widening and slimming compared with the classic Helmholtz square illusion [59].

The effect can be further amplified by introducing a central element (a red rectangle), which reinforces the accentuated directionality established by the previous figural conditions (Figure 3—third row). We propose to term this latter phenomenon the “tie effect”. These findings suggest potential applications in fashion design, where elements such as ties in men’s clothing, or necklaces, pendants, and necklines in women’s fashion, may serve to create illusions of elongation and slimming, thereby enhancing the perceived elegance of body and posture (see Figure 3—fourth row).

**Figure 4:**
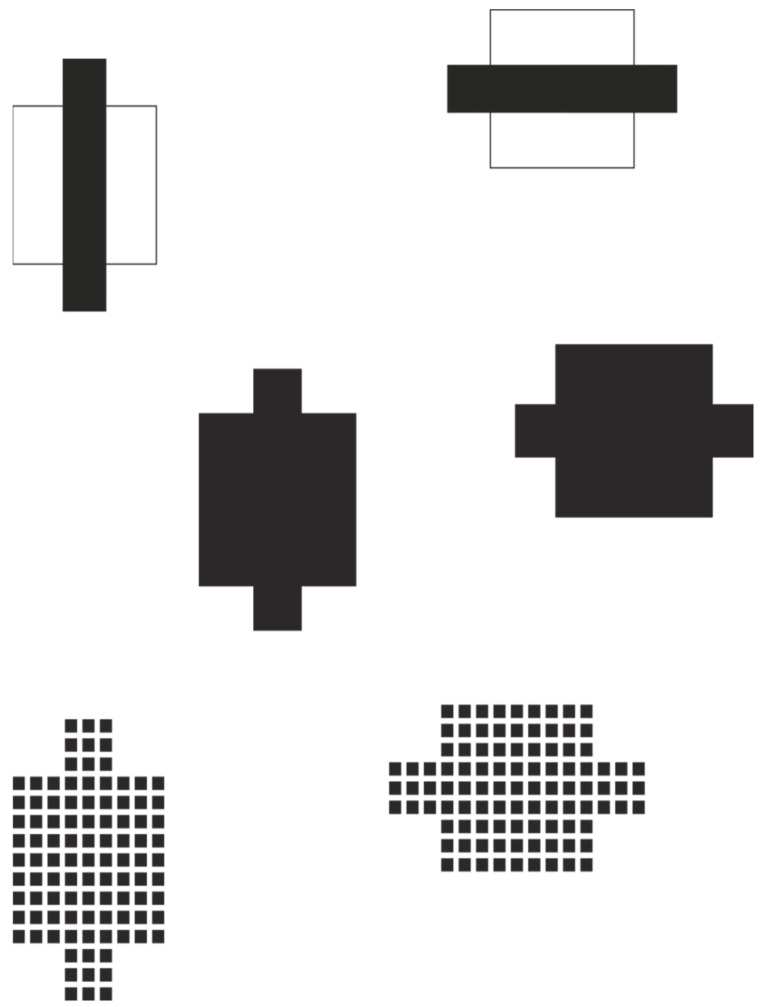
The presence of vertical and horizontal strips induces the illusory deformation of the square in the direction defined by the strip.

Further examples of the tie effect, even more pronounced than those in Figure 3, can be observed in the following conditions (Figure 4). The introduction of a vertical or horizontal strip (either modal or amodal) induces a deformation of the square in the direction defined by the strip. The strength of this induced deformation becomes apparent when analytically comparing the geometrically identical squares [60].

These examples contribute to our understanding of how simple geometric elements can significantly influence shape perception, and they may have implications for fields ranging from visual design to the study of camouflage in nature [1,61].

The tie effect and its relationship to attention present an intriguing area for consideration. While the tie effect is primarily a bottom-up perceptual phenomenon based on low-level visual features, it interacts significantly with attentional processes. The accentuation created by the tie effect can be viewed as a form of visual saliency that automatically captures attention [14]. However, unlike purely bottom-up attentional capture, the tie effect creates a sustained perceptual illusion that persists even under scrutiny. This suggests an interaction between bottom-up saliency and top-down attentional control [62,63]. The tie effect may serve to guide attention along specific directional axes, potentially facilitating faster processing of form information aligned with these axes (cf. [64]). Moreover, the ability of observers to perceive the illusory elongation or widening while simultaneously being aware of the true geometric properties of the stimulus demonstrates the flexibility of attention in switching between different levels of perceptual interpretation [65]. This interplay between accentuation and attention in the tie effect illustrates the complex dynamics of visual perception, where low-level feature accentuation can influence higher-level attentional processes and shape our conscious percepts.

The last illusory phenomena, while not immediately apparent, are distinctly perceptible. Once observed, they demonstrate remarkable persistence, becoming challenging or even impossible to suppress or modify. This attentional effect is particularly salient in the domains of design, art, and human fashion. Despite a lack of comprehensive understanding of all potential emergent effects, individuals often possess an intuitive capacity to perceive how accents influence their apparent physique, elegance, masculinity, femininity, aggressiveness, or serenity. These significant perceptual qualities, which can be diverse or even antithetical, are frequently modulated by subtle variations, details, minor dissimilarities, and delicate accentuations [66].

In some instances, these effects involve chromatic accentuations, such as those achieved through the application of lipstick or nail polish, which are associated with enhanced perceptions of femininity and sensuality [67]. They extend to all accentuations designed to augment sexual dimorphism, which is comparatively subtle in human beings. Sexual dimorphism refers to the differential physical characteristics between males and females of the same species, encompassing traits such as body size, coloration, ornamentation (e.g., antlers, feathers), or behaviors not directly linked to reproductive organs [68]. This dimorphism, driven by sexual selection, emerges from evolutionary pressures related to mating strategies, survival, and reproduction [69].

These accentuations serve as potent attractors of attention, which is predominantly drawn to and guided by such dissimilarities. It can be posited that attention actively seeks, detects, and transitions between dissimilarities, as these represent the primary sources of information in the visual field [14]. This aligns with the information theory perspective on visual perception, where regions of high information content (i.e., areas of unpredictability or uniqueness) are prioritized for attentional resources [70].

**Figure 5:**
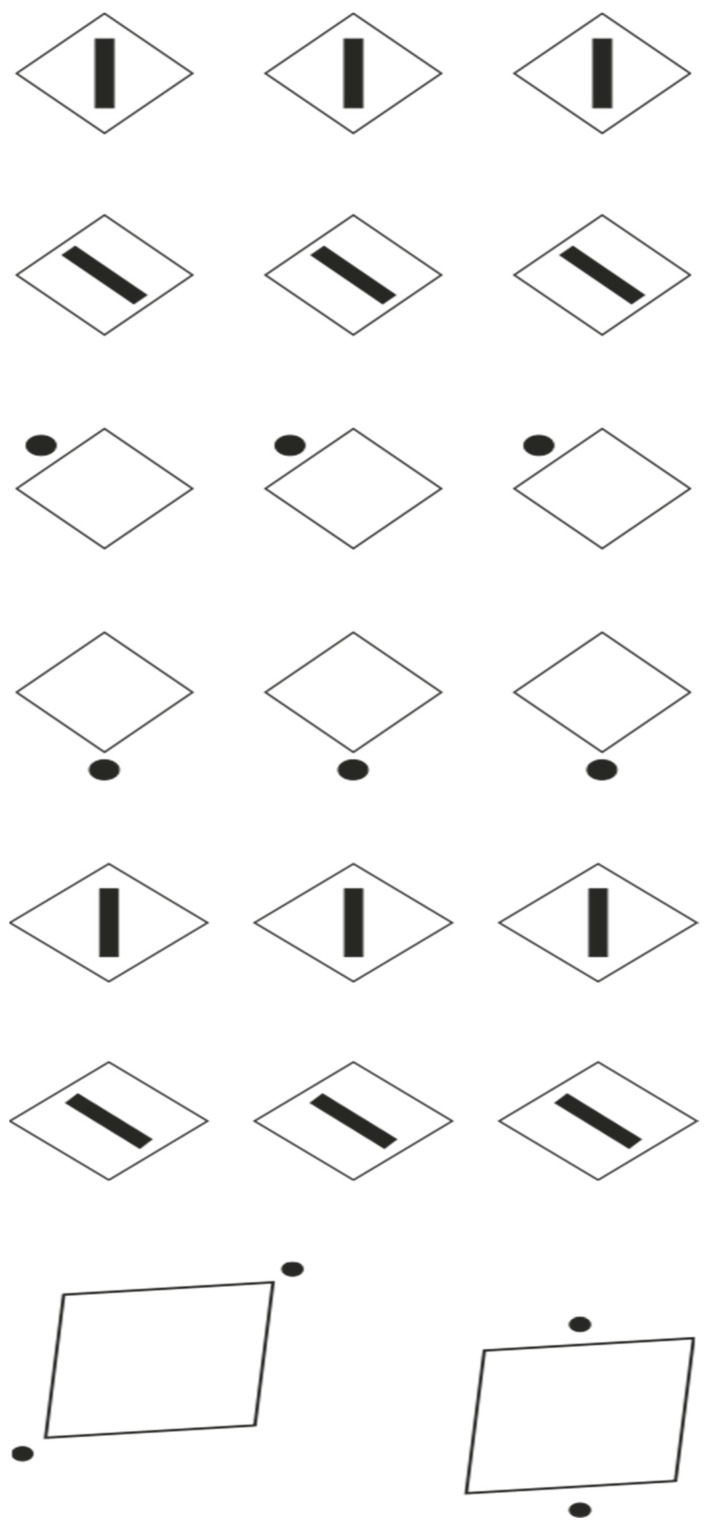
The presence of accents like strips and dots induces a strong illusory deformation of the geometrical shapes in the direction defined by the accentuation.

To further elucidate the perceptual effects achievable through accentuation, we examine additional examples. In Figure 5 (first four rows), the introduction of dissimilar elements to geometric squares, either internal (black rectangles) or external (dots), results in the accentuation of direction and position relative to the geometric shapes along their sides or angles. This gives rise to the rotated square–diamond effect: the large shapes in the first and fourth rows are perceived as diamonds, while those in the second and third rows appear as rotated squares [56]. Although geometrically identical, a square rotated 45 degrees and a diamond are phenomenologically distinct. The former emphasizes its square nature, appearing flattened along its sides, while the latter accentuates its angles and vertices. This demonstrates that a square comprises sides and angles that can be alternately emphasized through accentuation, suggesting that it inherently contains two potential forms, either of which can be highlighted through the principle of accentuation [12,23,24,54,55,71].

When geometric squares are replaced with rhombuses (Figure 5, fifth and sixth rows), accentuation leads to the perception of diamonds (fifth row) and parallelograms (sixth row), resulting in phenomenally distinct shapes despite their geometric equivalence. This effect persists when external dots replace internal rectangles (not illustrated). The varying degrees of distortion due to differently positioned dots, which accentuate different directions of the same figure, are observable in Figure 5—seventh row. Here, the overall deformation of the parallelogram is more pronounced in the first condition and partially compensated in the second.

From a phenomenological perspective, these results warrant reflection. While evident and immediate to most observers, uncovering the true geometric conditions is not challenging. Attention plays a crucial role in revealing the illusory nature of these percepts through analytical and comparative observation of the shapes and by focusing on their geometric orientation relative to primary spatial directions [64]. Attention, although initially drawn to accentuators that shape and spatially displace the object, emerges as a primary tool for unveiling illusions. Our subjects, after reporting their initial perceptions, acknowledged the illusory nature of these effects.

Attention facilitates a process of discovery and revelation of different perceptual interpretations, some more salient than others like prey–predator interactions in nature. For instance, antelopes have evolved acute vision and maintain constant vigilance for subtle environmental changes that might signal predator presence [41]. To survive, they must develop an awareness of potential illusions in their environment, such as camouflage, and discern between true and false perceptual inputs. This necessitates the ability to question their own perceptions and the act of perception itself. This involves a complex decision-making process: interpreting every minor environmental change as a danger signal would result in constant fighting or defensive behavior, while ignoring such changes could lead to falling prey to predators. The optimal strategy involves vigilant attention and awareness of what constitutes genuine danger, distinguishing it from background noise [72]. These observations highlight the critical role of stimulus-driven attention in both perceiving and overcoming perceptual illusions, demonstrating its importance in adaptive behavior and survival strategies in complex environments.

**Figure 6:**
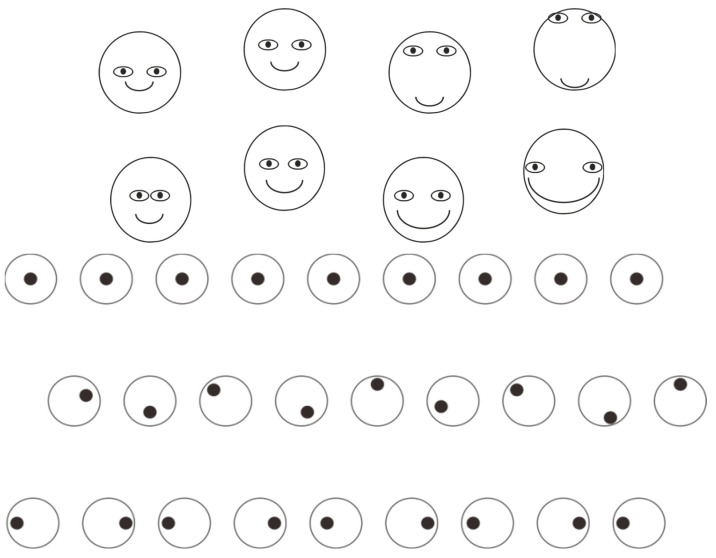
Illusory alterations in the perceived dimensions (vertically and horizontally) of the faces (first group). Perfect alignment of dots and circles, apparent misalignment of the circles, and horizontal convergence or divergence of the circles in pairs (second group).

Other configurations that appear markedly distinct upon initial inspection, such as the emoji faces depicted in Figure 6—first group, demonstrate that a progressive shift of the eyes and mouth along the vertical or horizontal axis alters a substantial illusory alteration in the perceived dimensions of the face, despite the fact that in all instances the face is comprised of an identical circumference. A comparative analysis of the first and last faces in both rows reveals an immediately discernible difference in the perceived width of the circle, which undergoes increasing vertical deformation in the first row and horizontal deformation in the second.

Consider how phenomenally significant small differences and relative distances are in the perception and aesthetics of the human face, particularly in the case of female makeup, the presence of imperfections or distortions, or cosmetic surgery. The relevance of the various effects of accentuators is also evident in the choice of accessories to enhance the aesthetics of the face, such as glasses, earrings, makeup itself, the color of lipstick, eye makeup, blush, eyelashes, and eyebrows, as well as the style of haircuts. The role of accentuators in all these cases is crucial, just as the role of attention is fundamental in detecting all the expressive nuances highlighted by various types of accents.

Figure 6—first group suggests an additional illusory phenomenon involving misalignment and variation in relative distance, as illustrated in Figure 6—second group. This group presents three rows of small circles, each containing an internal dot. In the first row, which functions as a control condition, the dot is centrally positioned within each circle. In the second row, the dots are vertically displaced, occupying either higher or lower positions, but consistently remaining within the confines of the circles. The third row introduces variation in the horizontal inter-dot distances. The perceptual outcomes are as follows: perfect alignment of dots and circles in the first row, apparent misalignment of the circles in another row, and finally, a perceived horizontal convergence or divergence of the circles in pairs in the third row, thereby altering the perceived relative distance between them. In all these cases, accent transforms the visual structure of the perceptual field.

**Figure 7:**
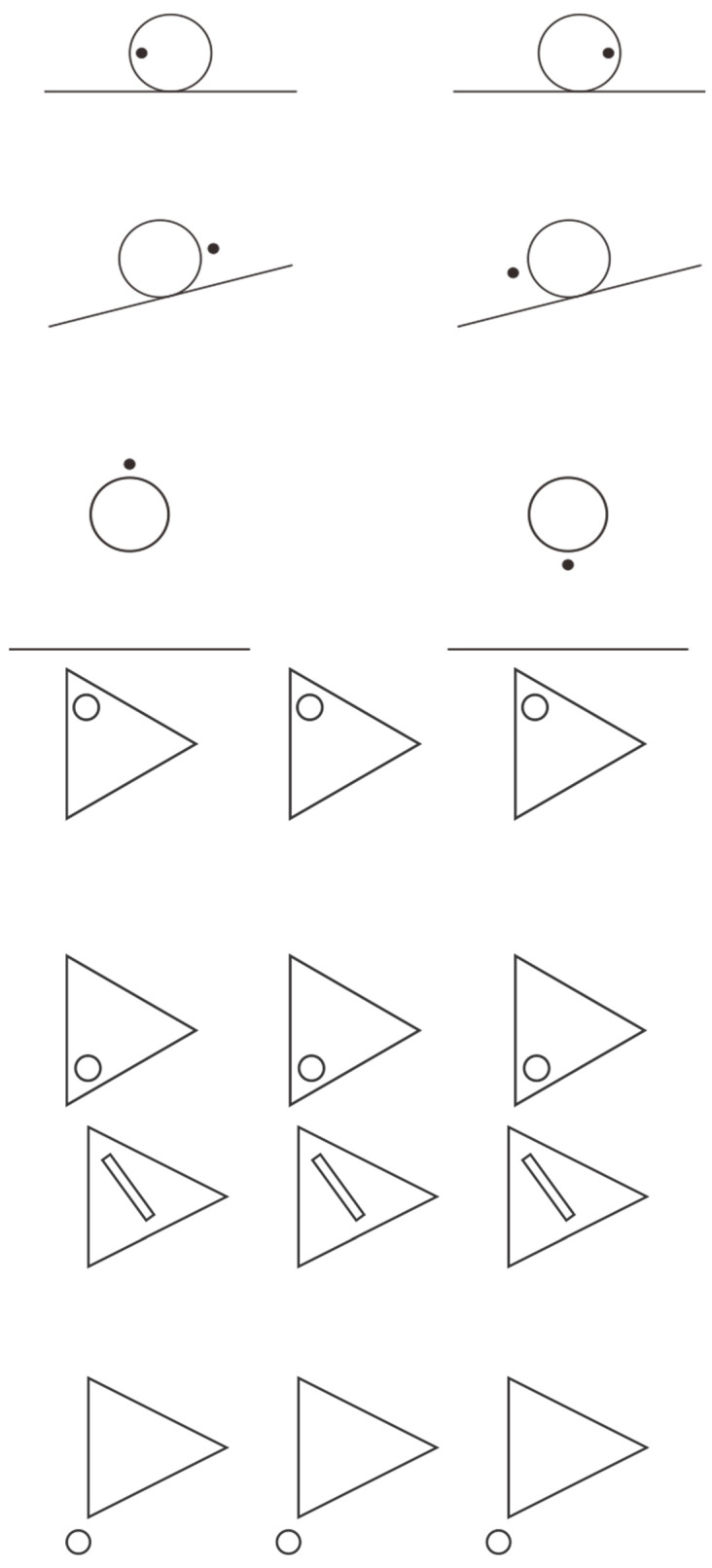
The ball is following the dot (first group). Despite the configural orientation effect indicating a rightward direction, the equilateral triangles are perceived as pointing upward in the first row and downward in the second (second group). Isosceles triangles appear as scalene triangles due to accentuation (third group).

More broadly, the enclosing elements, be they circles or squares, appear to be displaced in the direction defined by the dot. This principle enables the generation of additional illusory phenomena, as exemplified in Figure 7—first group, where the presence of a dot determines the perceived direction of motion for a stationary ball. The illusion suggests that the ball is following the dot, rather than the inverse. In the first row, the ball appears to roll leftward in the initial instance and rightward in the second. The second row demonstrates perceived upward and downward motion, respectively. In the third row, the ball seems to ascend or descend in accordance with the dot’s position.

This observation implies that the accent can also influence the orientation and the pointing, even contradicting the configural orientation effect studied by Attneave [73] and Palmer [74] (see also [23,24,54]). This effect demonstrates that the arrangement of three equilateral triangles determines their perceived pointing direction based on the overall configuration. While this could be interpreted as an accentuation effect resulting from their grouping, the phenomena presented earlier suggest that accentuation is predominantly a local rather than global phenomenon, operating within a broader reference system. This is readily observable in the conditions illustrated in Figure 7—second group, where, despite the configural orientation effect indicating a rightward direction, the equilateral triangles are perceived as pointing upward in the first row and downward in the second.

Further exploration of this phenomenon yields additional insights. By substituting isosceles triangles for equilateral ones, oriented synergistically according to the configural orientation effect, we can enhance the phenomenon’s complexity. If the accentuation of each triangle in a different direction is sufficiently pronounced, we would anticipate a perceived deformation of the isosceles triangles, transforming them into apparent scalene triangles. This outcome is clearly demonstrated in Figure 7—third group. Consequently, beyond the directional effect, a change in shape occurs, analogous to the previously described perceptual switch between a rotated square and a diamond. Again, accent transforms the structure and figural meaning of a shape. These types of accentuations are particularly significant in building—architecture, design, and art—where disruptions in weight, orientation, and alignment play a fundamental role in determining the configuration of the accentuated object, as well as all its expressive and artistic qualities. The artist’s skill lies in their attentiveness to such specific accentuators.

Regarding the interplay between accentuation and attention, it is pertinent to revisit the previously proposed concept that a square inherently contains at least two potential forms. Accentuation serves as an effective tool for bringing one form or the other to perceptual prominence. When one form emerges, the alternative remains implicit or entirely obscured.

**Figure 8:**
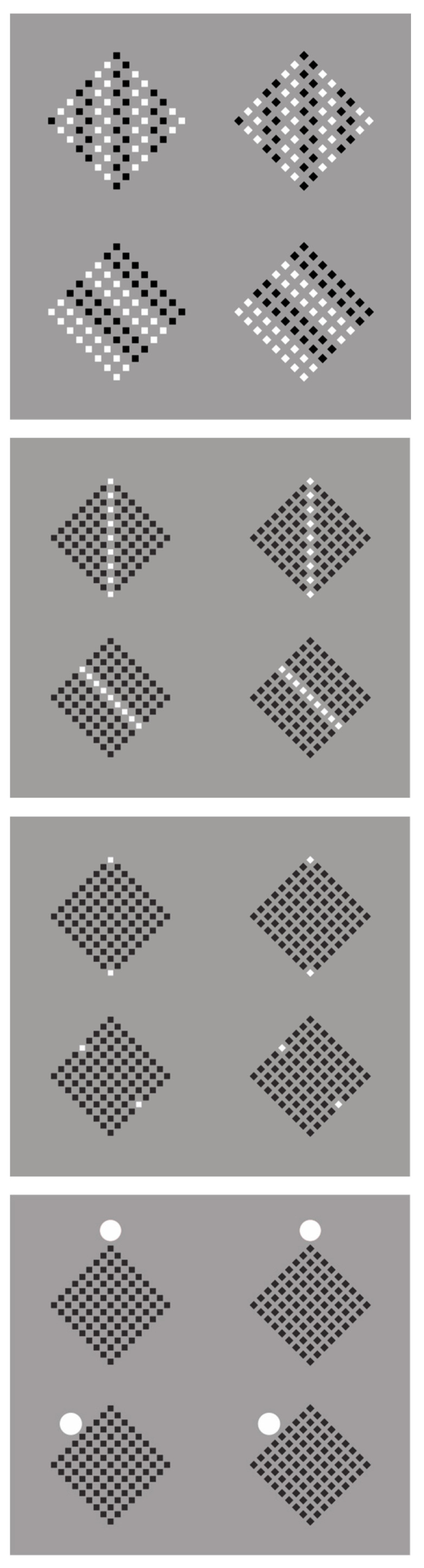
First and second group of figures: squares perceived as squares and diamonds as diamonds (1st row of the two groups); squares as diamonds and diamonds as rotated squares (2nd row of the two groups). Third and fourth group: the role of accentuation becomes more and more salient in both groups, where the perceptual organization based on similarity is gradually reduced to the limiting case of an external circle that is totally dissimilar and independent from the set of elements.

The double shape effect within the square is further exemplified in Figure 8—first group, where the rotated square–diamond phenomenon encompasses not only the overall matrix of elements (in this instance, composed of small squares) but also the individual elements themselves.

Specifically, in the top row, the square elements are perceived as squares (left), and the diamond elements are perceived as diamonds (right). These elements are embedded within a diamond-shaped whole, which is perceived as a large diamond in both conditions. This outcome aligns with expectations, as the direction of similarity accentuates the sides of the squares on the left and the angles of the diamonds on the right, both at the level of individual elements and overall shapes [56].

In the bottom row, a perceptual inversion occurs: the squares are perceived as diamonds (left), and the diamonds are perceived as rotated squares (right). Correspondingly, the global diamond shapes in both conditions are perceived as large rotated squares. In these instances, the direction of grouping highlights the attribute that contrasts with the geometrical shape, angles instead of sides, and sides instead of angles. In these cases, the interaction between local and global configurational processes constrains the perceptual result [75].

These results underscore the complex interplay between bottom-up processes of accentuation and top-down attentional mechanisms in shaping our visual percepts. The ability to switch between different interpretations of the same stimulus (e.g., seeing a square as a diamond and vice versa) illustrates the role of attention in resolving perceptual ambiguities and highlights the constructive nature of visual perception [76].

Moreover, this double-shape effect exemplifies the hierarchical nature of visual processing, where local features interact with global configurations to determine the final percept. This interaction between local and global processing is a fundamental aspect of visual cognition and has been extensively studied in the context of hierarchical stimuli [75,77,78].

The role of accentuation, as an independent principle from the classical Gestalt ones, becomes more and more salient in Figure 8—second, third, and fourth row, where the perceptual organization based on similarity is gradually reduced to the limiting case of an external circle that is totally dissimilar and independent from the set of elements.

In all these instances, the rotated square–diamond effect remains as pronounced as in the previous conditions. This observation provides compelling evidence for the potency of accentuation in shaping visual perception, even when reduced to minimal cues. This occurs because accents, when visible, immediately attract and capture attention. Attention in virtue of accents rearranges the structure of the perceptual object.

The external circle, despite its dissimilarity and independence from the internal elements, serves as a powerful organizing principle for the perception of the overall shape. The robustness of this effect across various configurations (from complex arrangements to this simplified case) underscores the fundamental nature of accentuation in visual perception. It suggests that accentuation might be an early, pre-attentive process that sets the stage for subsequent attentional and cognitive operations [79]. This fits into the experimental evidence that local structures are processed unconsciously and without awareness, while global shape assignment and structure integration is a set of processes accompanied by visual awareness [80].

We now explore how the effects of accentuation extend to shapes beyond the square, circle, and triangle, which we have focused on thus far. We ask the following: If a square contains within it two potential forms that can be accentuated by a simple dot, what happens to an irregular quadrilateral with four different sides and four distinct angles? How many internal shapes can be accentuated given the variability in its shape attributes? Based on our findings, we can infer that the existing diversity or dissimilarity can give rise to multiple distinct shapes, resulting in a total of eight possible forms.

**Figure 9:**
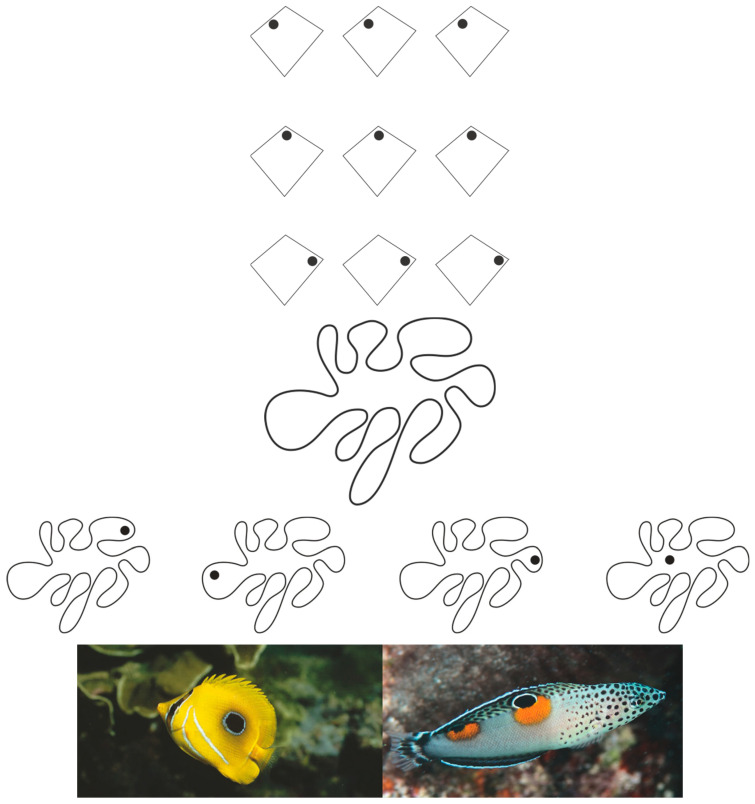
First, second, and third group: Trapezoids, rhomboids, and other shapes within the same geometrical quadrilateral (first group). Different organic shapes from accentuation (second group). Ocelli in the markings of fishes enhance adaptive fitness by altering shape and expressive qualities (third group).

In the first group of Figure 9, for simplicity, three of these eight possible forms are displayed, aligned in contrast to the configural orientation effect. This further emphasizes the strength of the accentuation principle and its ability to locally attract attention.

As can be easily observed, the elements in the first row appear as trapezoids, in the second as rhomboids, and in the third as shapes that are distinct but not easily identifiable. It is as though accentuation creates a kind of camouflage, making each shape different and nearly unrecognizable compared with the typical geometric form.

The phenomenon becomes even more apparent when we move from a quadrilateral to a more complex irregular shape, like the one illustrated in the second group of Figure 9, which initially appears as an irregular, undulating amoeboid shape, without any part being particularly prominent or more salient than others, except for the left side, which is wider than the rest. However, at first glance, nothing seems to stand out significantly, with attention shifting from one part to another without lingering on any single component. When a dot is added, the accentuated region emerges in the foreground, influencing the perception of all other parts in relation to it (second row). With the addition of the dot, the irregular shape transforms into a sort of organism, seemingly moving in the direction of the accentuated region, as seen in Figure 7. Simultaneously, all other components of the organism adapt to the characteristics of the accentuated part, making the illustrated shapes appear distinct from one another across multiple attributes, not just in terms of expression and expressive qualities.

Just as ties, necklaces, necklines, and other pendants elongate and slim the human body, natural accentuators, such as ocelli found in the markings of many organisms, starting with butterflies, enhance adaptive fitness by altering shape and expressive qualities, similar to the amoeboid form previously described. These accentuators serve vital biological functions, such as facilitating predation by altering the organism’s appearance to deceive prey or evade predators (see Figure 9-third group). Moreover, these modifications play a crucial role in sexual selection by enhancing traits that are attractive to potential mates [12]. This strategic use of accentuators for survival and reproduction is consistent with principles of perceptual organization and visual camouflage, as discussed in the introduction.

**Figure 10:**
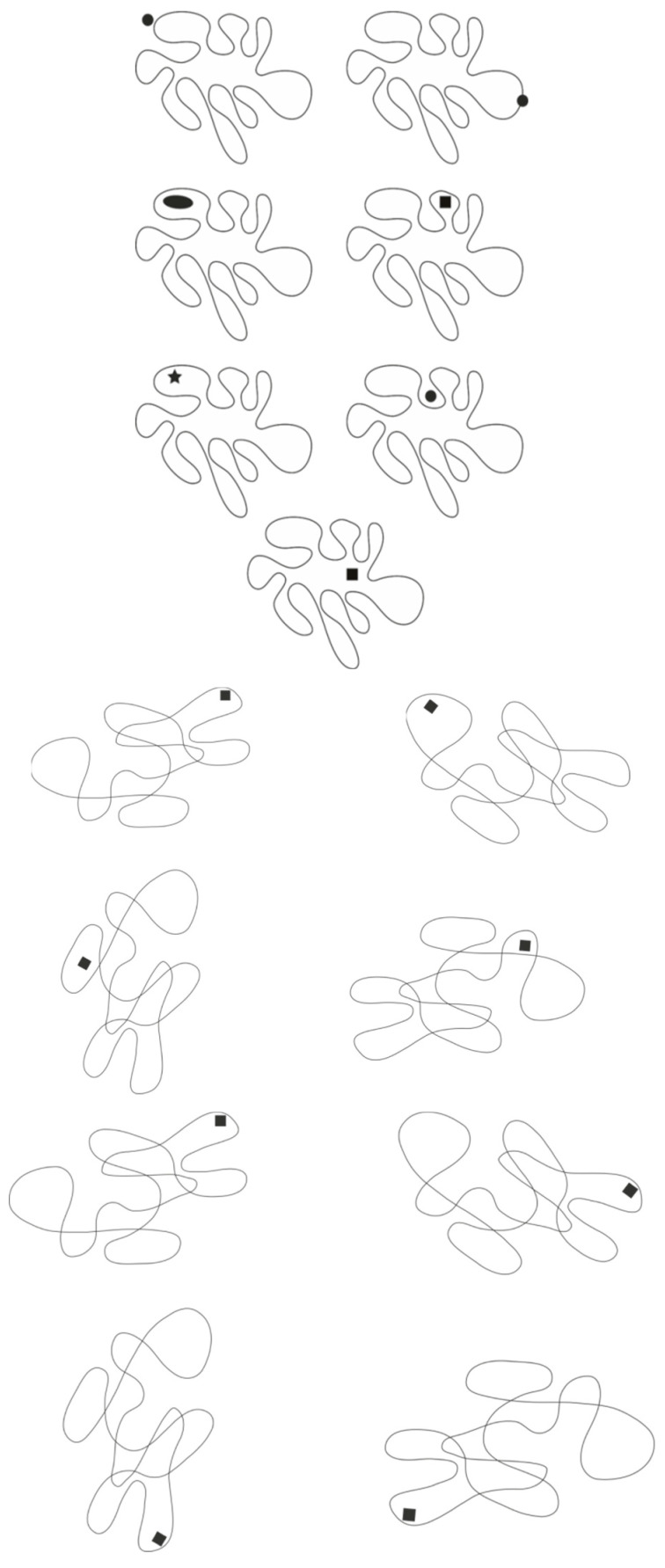
First, second, and third group: Even if accents do not resemble eyes yet still function similarly to the dot (first group). Accents can not only render the same irregular shape unrecognizable (second group) but also make it easily recognizable as the same rotated shape (third group).

We might ask how many possible shapes can be accentuated with this kind of object. Undoubtedly, there are many more than those found in the square or irregular quadrilateral. One could also question whether the dot functions like an eye, attributing head-like characteristics to the region where it is placed, with the body following. It is likely that this attribution occurs, but not because the dot resembles an eye. This can be demonstrated by the examples shown in the first group of Figure 10, where the accents do not resemble eyes yet still function similarly to the dot.

The use of accents can not only render an irregular shape unrecognizable, as illustrated in the second group of Figure 10, where the four shapes correspond to the same figure progressively rotated but also make it easily recognizable as the same rotated shape, as demonstrated in the third group of Figure 10.

**Figure 11:**
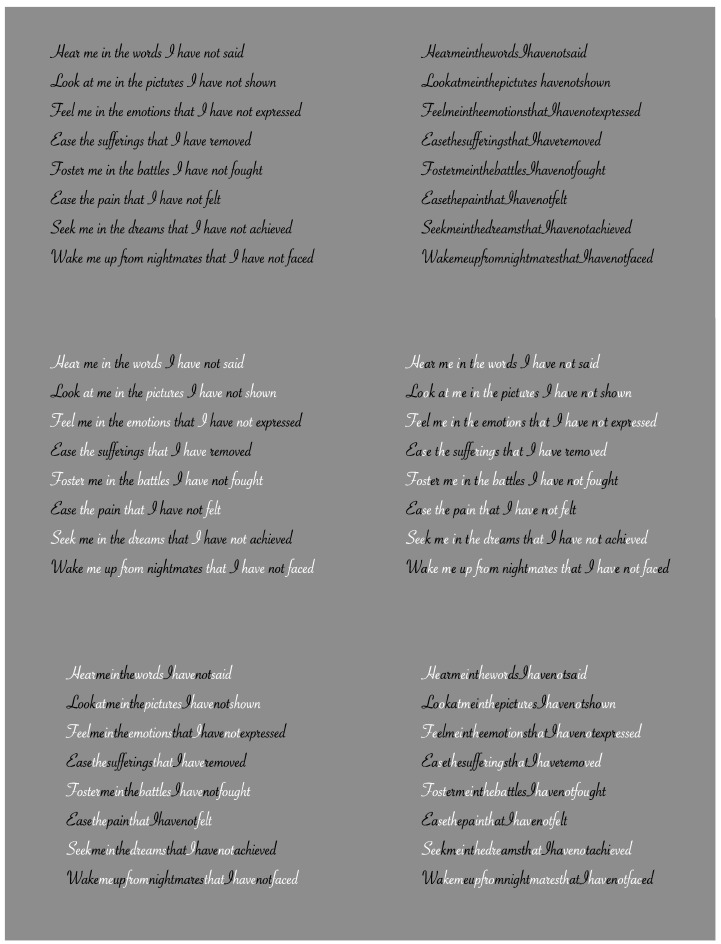

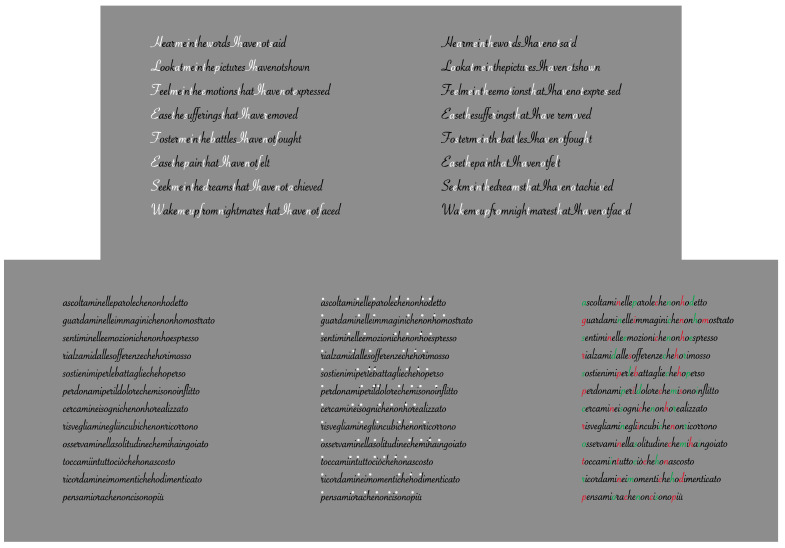
Accents can either facilitate or hinder the process of reading a text.

One final effect, though no less important, is that accents can either facilitate or hinder the reading process. In Figure 11, several conditions are shown in which accentuating entire words or specific letters in different positions through contrast polarity, whether or not spaces between words are present, can either ease or complicate the reading process, affecting reading speed or increasing the likelihood of errors. Similar effects are achieved using different colors or placing dots above individual letters. In all these cases, the role of accents as attractors and focal points for attention is evident.

The reader is encouraged to try reading the text in all presented combinations to observe this effect. The role of chromatic accentuation and contrast polarity in the reading process can be demonstrated with dyslexic individuals, showing improvements in reading speed and accuracy, as well as enhanced comprehension [53,81,82].

In the next section, we explore the detailed relationship between accents and attention, and more broadly, the link between perceptual organization driven by the principle of accentuation and consciousness, addressing more explicitly the questions posed in the introductory section of this work.

## 4. Discussion

The results of this study demonstrate the critical role of accentuation in perceptual organization, specifically in relation to stimulus-driven attention. The findings extend the classical Gestalt principles by introducing dissimilarity as a complementary attribute to similarity in determining how visual elements are grouped and perceived. Our results show that dissimilarity, through the mechanism of accentuation, plays an equally important role in guiding attention and revealing inner visual attributes. This is also supported by preliminary results from several eye-tracking experiments, which indicate that dissimilar, accentuated, and heterogeneous configuration induces a structured pattern of gaze transfer, whereas a visual configuration with a homogenous similarity-based grouping leads to a rather heterogeneous structure of eye movements [83].

One of the most intriguing aspects of our findings is the relationship between accentuation and perceptual illusions. The figures illustrate that small, subtle differences in accentuation can lead to significant perceptual distortions, such as the perceived elongation, contraction, or reorientation of objects. These illusions highlight perceptual organization beyond Gestalt grouping and suggest that attention, drawn to dissimilar elements, is not merely passive but actively shapes the interpretation of visual stimuli.

More particularly, accentuation represents a powerful tool in the perceptual organization process, serving not only to highlight key elements within a visual scene but also to influence the perceived structure of objects. By recognizing dissimilarity as a principle that works in concert with similarity, this study opens up new avenues for understanding how attention and perception are intertwined in the complex task of interpreting the world around us.

Our findings suggest that accentuation shapes initial perceptual organization, influencing how elements are grouped and perceived as wholes, and modulates the apparent shape, orientation, and relationships between visual elements on a local level. Attention, on the other hand, acts as a selective mechanism, focusing cognitive resources on specific aspects of the visual scene on a more global level of processing [80,84]. It facilitates detailed analysis and comparison of visual elements and enables the detection of illusions and the ability to switch between different perceptual interpretations and attentional control [85]. Accentuation is one of the strong evolutionary drivers of attentional selectivity.

The advantages of accentuation are evident in its rapid processing capabilities. Operating quickly, potentially before full attentional deployment, accentuation allows for fast initial scene interpretation. It automatically draws focus to potentially important visual features or discontinuities, like a bottom-up attentional capture [62]. As seen in our stimuli, accentuation can dramatically alter perceived shapes and orientations, potentially enhancing certain visual features for adaptive purposes. This ability plays a crucial role in both hiding and highlighting features in nature, aiding in survival and reproduction, as discussed in our introduction.

However, accentuation is not without its drawbacks. As demonstrated in multiple figures, it can lead to perceptual illusions, potentially misrepresenting the true nature of visual stimuli. Furthermore, accentuation appears to be largely stimulus-driven and less flexible than attentional processes. While it excels at highlighting features, it does not provide the detailed analysis that attention can offer [79] (for overviews see also [86,87]).

Attention, in contrast, offers greater flexibility. It can be voluntarily directed, allowing for the exploration of different aspects of a visual scene, a characteristic central to the concept of endogenous attention [64] (on the flexibility of attention see also [88]). This flexibility enables fine-grained analysis of visual elements, as seen in the ability to detect the illusory nature of accentuated shapes. As noted in our discussion of several stimuli, attention plays a crucial role in uncovering the true geometric properties of shapes, beyond their accentuated appearance. It also allows for switching between different interpretations of ambiguous stimuli, as seen in the square–diamond illusions, similar to the role of attention in binocular rivalry [89].

Nevertheless, attentional processes are not without their limitations. They require more cognitive resources than automatic accentuation and typically involve slower processing, as evidenced by studies on the attentional blink [90].

The results presented demonstrate a complex interplay between accentuation and attention. While accentuation provides an initial perceptual organization, attention can either reinforce this organization or reveal alternative interpretations. This dynamic interaction suggests a two-stage process of visual perception: an initial stage of rapid, automatic accentuation-based organization, followed by a secondary stage of more detailed, flexible attentional analysis (e.g., [19,79,80,91]). We agree that perceptual organization and attentional selectivity constrain one another; however, the initial effect of accentuation occurs pre-attentively and without awareness (see [19,20,80]). The accent seems to prepare a primary perceptual entrance for attentional processing.

The implications of this research are far-reaching. The interplay between accentuation and attention may be crucial in developing a full understanding of perceptual awareness and consciousness [92]. Understanding how accentuation guides initial perception could inform more effective visual search strategies [93]. The principles of accentuation could be applied in fields such as graphic design, user interface design, and visual communication to guide attention effectively. Furthermore, the relationship between accentuation and attention might provide insights into attentional disorders and visual perception deficits, opening avenues for clinical applications [94] and research on neurodegenerative impairments (e.g., [95]).

Investigating the neural mechanisms underlying accentuation and its interaction with attentional networks could provide valuable insights into brain function [96]. Exploring the time course of accentuation effects and how they transition to attentional processing would enhance our understanding of the temporal dynamics of perception. Examining how individual differences in perceptual processing might influence the balance between accentuation and attention could reveal important variations in cognitive processing. Finally, extending the study of accentuation to other sensory modalities and exploring cross-modal interactions could broaden our understanding of sensory processing as a whole [97].

The relationship between accentuation, attention, and consciousness presents a fascinating avenue for exploration in cognitive neuroscience. Our findings suggest that accentuation may play a crucial role in the early stages of conscious perception, potentially influencing what information becomes available to consciousness, which emphasizes the importance of information integration and availability in conscious experience [98,99].

The interplay between accentuation and attention observed in our study may shed light on the mechanisms of conscious access. For instance, the ability of attention to reveal the illusory nature of accentuated perceptions suggests that attention might serve as a gatekeeper for conscious awareness, allowing for more detailed and accurate representations to enter consciousness [100]. This supports the notion of a hierarchy in perceptual processing, where initial, rapid accentuation-based percepts can be superseded by more refined, attention-driven conscious perceptions.

Moreover, the phenomenon of accentuation could provide insights into the ongoing debate about the richness of conscious experience [101]. The fact that accentuation can dramatically alter perceived shapes and orientations without immediate conscious awareness of this alteration raises questions about the nature of phenomenal consciousness versus access consciousness. It suggests that our visual experience might be richer and more detailed at a pre-conscious level than what is typically available for cognitive access and reporting.

Furthermore, the observed effects of accentuation on reading hint at the potential influence of primary perceptual processes on higher-order cognitive functions that are typically associated with consciousness. This raises intriguing questions about the relationship between perceptual organization, language processing, and conscious thought.

Finally, integrating the concept of accentuation into theories of consciousness offers a novel perspective on how our brains construct our conscious experience of the world. It highlights the complex interplay between bottom-up perceptual processes and top-down attentional modulation in shaping what we become aware of. As we continue to unravel the neural basis of consciousness, considering the role of accentuation alongside attention may prove crucial in developing a comprehensive understanding of how our subjective experience of the world emerges from neural activity.

The relationship between accentuation, attention, and information processing emerges as a central theme in our findings. From an information theory perspective, accentuation can be viewed as a mechanism that increases local information content by introducing dissimilarities or discontinuities in the visual field. These areas of high information content naturally attract attention, aligning with the idea that attention serves to optimize information gathering [70,86].

Our results suggest that accentuation acts as an initial filter, supporting attentional selectivity and highlighting potentially relevant information for further attentional processing. This two-stage process (rapid, automatic accentuation followed by more detailed attentional analysis) may represent an efficient strategy for managing the vast amount of visual information available at any given moment. The interplay between accentuation and attention also offers insights into how the visual system balances the need for rapid scene interpretation with the requirement for accurate object recognition. Accentuation provides quick, albeit sometimes illusory, interpretations that may be sufficient for many tasks. Attention then allows for more detailed scrutiny when needed, potentially correcting misinterpretations caused by accentuation. This dynamic relationship might be understood as a trade-off between speed and accuracy in information processing, with implications for our understanding of visual consciousness and decision-making processes.

Moreover, our findings on the evolutionary aspects of accentuation in camouflage and sexual selection underscore how these perceptual mechanisms may have evolved to optimize the detection and processing of biologically relevant information. The ability to rapidly detect dissimilarities (through accentuation) and selectively focus on them (through attention) could provide significant adaptive advantages in predator–prey interactions and mate selection scenarios, effectively reducing entropy in complex visual environments to extract survival-critical information (on the neural basis of visual selective attention see [102]).

In conclusion, in the introductory section, we posed several critical questions that we now shortly address in light of our results. What is the nature of accentuation? Accentuation is a pre-attentive mechanism that highlights salient features or discontinuities in the visual field. It shapes initial perceptual organization, influencing how elements are grouped and perceived as wholes, and modulates the apparent shape, orientation, and relationships between visual elements. Accentuation operates rapidly and automatically, setting the stage for subsequent attentional processing. A question for future work would be to examine the real-time dynamics of attentional processes, e.g., in eye tracking, and compare the results with object vs. space-based approaches of attention [21,71,83,103].

What is the role of attention in the perceptual organization due to accentuation and camouflage? We found that attention acts as a flexible, selective mechanism that can either reinforce or override accentuation-based percepts. It plays a crucial role in unveiling illusions and allowing for detailed analysis of visual stimuli. Attention facilitates the discovery and revelation of different perceptual interpretations, some more salient than others. In the context of camouflage, attention enables the discrimination between true and false perceptual inputs, demonstrating its importance in adaptive behavior and survival strategies.

What is the relationship between the concept of accentuation and the notion of information? Our results suggest that accentuation increases local information content. From an information theory perspective, accentuation can be viewed as a mechanism that optimizes information gathering by highlighting potentially relevant areas for further processing.

To what extent are perceptual organization and attentional mechanisms related? Our study reveals a two-stage process of visual perception: an initial stage of rapid, automatic accentuation-based organization, followed by a secondary stage of more detailed, flexible attentional analysis. This demonstrates a close relationship between perceptual organization and attentional mechanisms, where accentuation provides an initial structure that attention can then explore, reinforce, or modify.

How does species evolution manipulate the principles of perceptual organization and attentional biases to deceive other organisms while simultaneously evolving mechanisms to avoid deception? The findings on camouflage and sexual selection suggest that accentuation and attention have evolved as complementary mechanisms to optimize the detection and processing of biologically relevant information. The ability to rapidly detect dissimilarities (through accentuation) and selectively focus on them (through attention) provides significant adaptive advantages in predator–prey interactions and mate selection scenarios. This dual system allows organisms to both create effective camouflage and see through the camouflage of others, demonstrating the evolutionary arms race in perceptual systems.

How do accentuation and camouflage influence the perception of ambivalence, uncertainty, awareness, and the relationship between perceived danger and objective reality? Our results indicate that accentuation plays a role in determining what information becomes available to consciousness, influencing the contents of visual awareness. The interplay between accentuation and attention can lead to varying levels of ambivalence and uncertainty in perception, as demonstrated by the illusory effects we observed. This relationship affects the ability to discern between true and false perceptual inputs, which is crucial for accurately assessing danger in natural environments.

Finally, these findings contribute to a more comprehensive understanding of visual perception, attentional processes, and consciousness. They highlight the complex interplay between bottom-up perceptual and attentional processes and top-down attentional modulation in shaping our subjective experience of the world. Our study opens new avenues for research in cognitive neuroscience, evolutionary biology, and applied fields such as design and clinical psychology, providing a foundation for further exploration of how our visual system constructs and interprets the world around us.

## Data Availability

The raw data supporting the conclusions of this article will be made available by the authors upon request.

## References

[1] Cott H.B. (1940). Adaptive Coloration in Animals.

[2] Stevens M. (2005). The role of eyespots as anti-predator mechanisms, principally demonstrated in the Lepidoptera. Biol. Rev..

[3] Kodandaramaiah U. (2011). The evolutionary significance of butterfly eyespots. Behav. Ecol..

[4] Ghose G.M., Harrison I.T. (2009). Temporal precision of neuronal information in a rapid perceptual judgment. J. Neurophysiol..

[5] Weiner K.F., Ghose G.M. (2014). Rapid shape detection signals in area V4. Front. Neurosci..

[6] Weiner K.F., Ghose G.M. (2015). Population coding in area V4 during rapid shape detections. J. Neurophysiol..

[7] De Bona S., Valkonen J.K., López-Sepulcre A., Mappes J. (2015). Predator mimicry, not conspicuousness, explains the efficacy of butterfly eyespots. Proc. Biol. Sci..

[8] Prudic K.L., Stoehr A.M., Wasik B.R., Monteiro A. (2015). Eyespots deflect predator attack increasing fitness and promoting the evolution of phenotypic plasticity. Proc. Biol. Sci..

[9] Pinna B., Reeves A. (2013). What is the purpose of color for living beings? Toward a theory of color organization. Psychol. Res..

[10] Pfennig D.W., Mullen S.P. (2010). Mimics without models: Causes and consequences of allopatry in Batesian mimicry complexes. Proc. Biol. Sci..

[11] Breuker C.J., Brakefield P.M. (2002). Female choice depends on size but not symmetry of dorsal eyespots in the butterfly Bicyclusanynana. Proc. Biol. Sci..

[12] Pinna B. (2021). La Percezione Visiva.

[13] Stevens M., Stubbins C.L., Hardman C.J. (2008). The anti-predator function of ‘eyespots’ on camouflaged and conspicuous prey. Behav. Ecol. Sociobiol..

[14] Itti L., Koch C. (2001). Computational modelling of visual attention. Nat. Rev. Neurosci..

[15] Desimone R., Duncan J. (1995). Neural mechanisms of selective visual attention. Annu. Rev. Neurosci..

[16] Endler J.A. (1981). An overview of the relationships between mimicry and crypsis. Biol. J. Linn. Soc..

[17] Ryan M.J., Cummings M.E. (2013). Perceptual Biases and Mate Choice. Annu. Rev. Ecol. Evol. Syst..

[18] Dall S.R., Giraldeau L.A., Olsson O., McNamara J.M., Stephens D.W. (2005). Information and its use by animals in evolutionary ecology. Trends Ecol. Evol..

[19] Kahneman D., Henik A., Kubovy M., Pomerantz J.R. (1981). Perceptual organization and attention. Perceptual Organization.

[20] Kimchi R. (2009). Perceptual organization and visual attention. Prog. Brain Res..

[21] Yantis S. (1992). Multielement visual tracking: Attention and perceptual organization. Cogn. Psychol..

[22] Albertazzi L., Wagemans J. (2015). Philosophical background: Phenomenology. Oxford Handbook of Perceptual Organization.

[23] Pinna B. (2010). New Gestalt principles of perceptual organization: An extension from grouping to shape and meaning. Gestalt Theory.

[24] Pinna B. (2010). What comes before psychophysics? The problem of ‘what we perceive’ and the phenomenological exploration of new effects. Seeing Perceiving.

[25] Pinna B., Albertazzi L., Albertazzi L., van Tonder G., Vishwanath D. (2011). From grouping to meaning. Perception Beyond Inference: The Information Content of Visual Processes.

[26] Pinna B., Reeves A. (2006). Lighting, backlighting and watercolor illusions and the laws of figurality. Spat. Vis..

[27] Pinna B., Sirigu L. (2011). The Accentuation Principle of Visual Organization and the Illusion of Musical Suspension. Seeing Perceiving.

[28] Kanizsa G. (1979). Organization in Vision.

[29] Kanizsa G. (1980). Grammatica del Vedere.

[30] Kanizsa G. (1991). Vedere e Pensare.

[31] Koffka K. (1935). Principles of Gestalt Psychology.

[32] Metzger W. (1963). Psychologie.

[33] Metzger W. (1975). Gesetze des Sehens.

[34] Spillmann L., Ehrenstein W.H., Chalupa L., Werner J.S. (2004). Gestalt factors in the visual neurosciences. The Visual Neurosciences.

[35] Wertheimer M. (1922). Untersuchungenzur Lehre von der Gestalt I. Psychol. Forsch..

[36] Wertheimer M. (1923). Untersuchungenzur Lehre von der Gestalt II. Psychol. Forsch..

[37] Wagemans J., Elder J.H., Kubovy M., Palmer S.E., Peterson M.A., Singh M., Von der Heydt R. (2012). A century of Gestalt psychology in visual perception: I. Perceptual grouping and figure–ground organization. Psychol. Bull..

[38] Kubovy M., van den Berg M. (2008). The whole is equal to the sum of its parts: A probabilistic model of grouping by proximity and similarity in regular patterns. Psychol. Rev..

[39] Pinna B., Conti L. (2021). Illusory figures: From logic to phenomenology. Psychol. Conscious..

[40] Pinna B., Conti L., Porcheddu D. (2021). On the role of contrast polarity in perceptual organization: A Gestalt approach. Psychol. Conscious..

[41] Caro T. (2005). Antipredator Defenses in Birds and Mammals.

[42] Caro T., Izzo A., Reiner R.C., Walker H., Stankowich T. (2014). The function of zebra stripes. Nat. Commun..

[43] How M.J., Zanker J.M. (2014). Motion camouflage induced by zebra stripes. Zoology.

[44] Chen L. (2005). The topological approach to perceptual organization. Vis. Cogn..

[45] Grossberg S., Mingolla E. (1985). Neural dynamics of form perception: Boundary completion, illusory figures, and neon color spreading. Psychol. Rev..

[46] Hojjatoleslami S.A., Kittler J. (1998). Region growing: A new approach. IEEE Trans. Image Process..

[47] Julesz B. (1981). A theory of preattentive texture discrimination based on first-order statistics of textons. Biol. Cybern..

[48] Julesz B. (1981). Textons, the elements of texture perception, and their interactions. Nature.

[49] Muir A., Warner M.W. (1980). Homogeneous tolerance spaces. Czech. Math. J..

[50] Pavlidis T., Liow Y.T. (1990). Integrating region growing and edge detection. IEEE Trans. Pattern Anal. Mach. Intell..

[51] Palmer S., Rock I. (1994). Rethinking perceptual organization: The role of uniform connectedness. Psychon. Bull. Rev..

[52] Pinna B., Porcheddu D., Skilters J. (2022). Similarity and dissimilarity in perceptual organization: On the complexity of the Gestalt principle of similarity. Vision.

[53] Pinna B., Porcheddu D., Deiana K. (2016). From Grouping to Coupling: A New Perceptual Organization in Vision, Psychology, and Biology. Front. Psychol..

[54] Pinna B. (2012). What is the meaning of shape?. Gestalt Theory.

[55] Pinna B. (2015). Directional organization and shape formation: New illusions and Helmholtz’s square. Front. Hum. Neurosci..

[56] Pomerantz J.R., Portillo M.C. (2011). Grouping and emergent features in vision: Toward a theory of basic Gestalts. J. Exp. Psychol. Hum. Percept. Perform..

[57] Künnapas T.M. (1955). An analysis of the ‘horizontal-vertical’ illusion. J. Exp. Psychol..

[58] Thompson P., Mikellidou K. (2011). Applying the Helmholtz illusion to fashion: Horizontal stripes won’t make you look fatter. I-Perception.

[59] Prinzmetal W., Gettleman L. (1993). Vertical-horizontal illusion: One eye is better than two. Percept. Psychophys..

[60] Spehar B., Halim L. (2016). Geometric illusions and context effects: The role of the visual field shape. J. Vis..

[61] Stevens M., Merilaita S., Stevens M., Merilaita S. (2012). Animal camouflage: Function and mechanisms. Animal Camouflage: Mechanisms and Function.

[62] Theeuwes J. (2010). Top–down and bottom–up control of visual selection. Acta Psychol..

[63] Kondo H.M., van Loon A.M., Kawahara J.I., Moore B.C. (2017). Auditory and visual scene analysis: An overview. Philos. Trans. R. Soc. Lond. B Biol. Sci..

[64] Carrasco M. (2011). Visual attention: The past 25 years. Vis. Res..

[65] Hochstein S., Ahissar M. (2002). View from the top: Hierarchies and reverse hierarchies in the visual system. Neuron.

[66] De Long M.R., Larntz K. (1980). Measuring visual response to clothing. Home Econ. Res. J..

[67] Russell R. (2009). A sex difference in facial contrast and its exaggeration by cosmetics. Perception.

[68] Fairbairn D.J., Blanckenhorn W.U., Székely T. (2007). Sex, Size and Gender Roles: Evolutionary Studies of Sexual Size Dimorphism.

[69] Andersson M.B. (1994). Sexual Selection.

[70] Bruce N.D., Tsotsos J.K. (2009). Saliency, attention, and visual search: An information theoretic approach. J. Vis..

[71] Krauze L., Skilters J., Delesa-Velina M., Pinna B., Krumina G. (2021). Gaze Parameters in the Analysis of Ambiguous Geometric Shapes. I-Perception.

[72] Dukas R., Kamil A.C. (2000). The cost of limited attention in blue jays. Behav. Ecol..

[73] Attneave F. (1968). Triangles as ambiguous figures. Am. J. Psychol..

[74] Palmer S.E. (1980). What makes triangles point: Local and global effects in configurations of ambiguous triangles. Cogn. Psychol..

[75] Kimchi R., Wagemans J. (2015). The perception of hierarchical structure. Oxford Handbook of Perceptual Organization.

[76] Tsal Y., Kolbet L. (1985). Disambiguating ambiguous figures by selective attention. Q. J. Exp. Psychol..

[77] Navon D. (1977). Forest before trees: The precedence of global features in visual perception. Cogn. Psychol..

[78] Kimchi R. (1992). Primacy of wholistic processing and global/local paradigm: A critical review. Psychol. Bull..

[79] Treisman A.M., Gelade G. (1980). A feature-integration theory of attention. Cogn. Psychol..

[80] Sabary S., Devyatko D., Kimchi R. (2020). The role of visual awareness in processing of global structure: Evidence from the perceptual organization of hierarchical patterns. Cognition.

[81] Pinna B., Deiana K. (2014). New conditions on the role of color in perceptual organization and an extension to how color influences reading. Psihologija.

[82] Pinna B., Deiana K. (2018). On the role of color in reading and comprehension tasks in dyslexic children and adults. I-Perception.

[83] Skilters J., Zarina L., Pinna B., Gintere M., Bartušēvica S., Umbrasko S., Platkājis A., Zeļģe L., Mednieks J., Ševčenko A. Visual accentuation constrains the structure of perceptual organization. Proceedings of the 46th Annual Conference of the Cognitive Science Society.

[84] Posner M.I., Petersen S.E. (1990). The attention system of the human brain. Annu. Rev. Neurosci..

[85] Corbetta M., Shulman G.L. (2002). Control of goal-directed and stimulus-driven attention in the brain. Nat. Rev. Neurosci..

[86] Evans K.K., Horowitz T.S., Howe P., Pedersini R., Reijnen E., Pinto Y., Kuzmova Y., Wolfe J.M. (2011). Visual attention. Wiley Interdiscip. Rev. Cogn. Sci..

[87] Knudsen E.I. (2007). Fundamental components of attention. Annu. Rev. Neurosci..

[88] Carlisle N.B. (2019). Focus: Attention science: Flexibility in attentional control: Multiple sources and suppression. Yale J. Biol. Med..

[89] Dieter K.C., Brascamp J., Tadin D., Blake R. (2016). Does visual attention drive the dynamics of bistable perception?. Atten. Percept. Psychophys..

[90] Raymond J.E., Shapiro K.L., Arnell K.M. (1992). Temporary suppression of visual processing in an RSVP task: An attentional blink?. J. Exp. Psychol. Hum. Percept. Perform..

[91] Wolfe J.M. (1994). Guided search 2.0: A revised model of visual search. Psychon. Bull. Rev..

[92] Koch C., Tsuchiya N. (2007). Attention and consciousness: Two distinct brain processes. Trends Cogn. Sci..

[93] Wolfe J.M. (2021). Guided search 6.0: An updated model of visual search. Psychon. Bull. Rev..

[94] Posner M.I., Rothbart M.K. (2007). Research on attention networks as a model for the integration of psychological science. Annu. Rev. Psychol..

[95] Rizzo M., Anderson S.W., Dawson J., Myers R., Ball K. (2000). Visual attention impairments in Alzheimer’s disease. Neurology.

[96] Kastner S., Ungerleider L.G. (2000). Mechanisms of visual attention in the human cortex. Annu. Rev. Neurosci..

[97] Spence C., Driver J. (2004). Crossmodal Space and Crossmodal Attention.

[98] Baars B.J. (2005). Global workspace theory of consciousness: Toward a cognitive neuroscience of human experience. Prog. Brain Res..

[99] Tononi G., Boly M., Massimini M., Koch C. (2016). Integrated information theory: From consciousness to its physical substrate. Nat. Rev. Neurosci..

[100] Dehaene S., Changeux J.P., Naccache L., Sackur J., Sergent C. (2006). Conscious, preconscious, and subliminal processing: A testable taxonomy. Trends Cogn. Sci..

[101] Block N. (2011). Perceptual consciousness overflows cognitive access. Trends Cogn. Sci..

[102] Chelazzi L., Della Libera C., Sani I., Santandrea E. (2011). Neural basis of visual selective attention. Wiley Interdiscip. Rev. Cogn. Sci..

[103] Yeshurun Y., Kimchi R., Sha’shoua G., Carmel T. (2009). Perceptual objects capture attention. Vis. Res..

